# Effectiveness and Cost-Utility Analysis of Different Doses of Irinotecan Plus Bevacizumab in Patients With Metastatic Colorectal Cancer: A Long-Term and Prospective Cohort Study

**DOI:** 10.3389/fonc.2022.756078

**Published:** 2022-03-14

**Authors:** Hon-Yi Shi, Yen-Cheng Chen, Ching-Wen Huang, Ching-Chun Li, Wei-Chih Su, Tsung-Kun Chang, Po-Jung Chen, Tzu-Chieh Yin, Hsiang-Lin Tsai, Jaw-Yuan Wang

**Affiliations:** ^1^Department of Healthcare Administration and Medical Informatics, Kaohsiung Medical University, Kaohsiung, Taiwan; ^2^Department of Business Management, National Sun Yat-sen University, Kaohsiung, Taiwan; ^3^Department of Medical Research, Kaohsiung Medical University Hospital, Kaohsiung, Taiwan; ^4^Department of Medical Research, China Medical University Hospital, China Medical University, Taichung, Taiwan; ^5^Division of Colorectal Surgery, Department of Surgery, Kaohsiung Medical University Hospital, Kaohsiung Medical University, Kaohsiung, Taiwan; ^6^Department of Surgery, Faculty of Medicine, College of Medicine, Kaohsiung Medical University, Kaohsiung, Taiwan; ^7^Graduate Institute of Clinical Medicine, College of Medicine, Kaohsiung Medical University, Kaohsiung, Taiwan; ^8^Division of General and Digestive Surgery, Department of Surgery, Kaohsiung Medical University Hospital, Kaohsiung Medical University, Kaohsiung, Taiwan; ^9^Department of Surgery, Kaohsiung Municipal Tatung Hospital, Kaohsiung Medical University, Kaohsiung, Taiwan; ^10^Center for Cancer Research, Kaohsiung Medical University, Kaohsiung, Taiwan; ^11^Center for Liquid Biopsy and Cohort Research, Kaohsiung Medical University, Kaohsiung, Taiwan; ^12^Pingtung Hospital, Ministry of Health and Welfare, Pingtung, Taiwan

**Keywords:** metastatic colorectal cancer, FOLFIRI, irinotecan dose escalation, quality of life, cost-utility analysis

## Abstract

**Objective:**

Patients with metastatic colorectal cancer (mCRC) had oncological benefits with irinotecan dose escalation of FOLFIRI regimen combined with bevacizumab according to *UGT1A1* genotypes in our previous study. In the current study, we performed a quality of life (QOL) outcome evaluation and cost-utility analysis of different irinotecan dose regimens in patients with mCRC.

**Materials and Methods:**

With inverse probability-of-treatment weighting (IPTW) matching on all covariates, 75 patients with dose escalation of irinotecan (study group) and 121 patients with the recommended dose of irinotecan (control group) were recruited between October 2015 and December 2019. The QOL outcome measures were Functional Assessment of Cancer Therapy-Colorectal, Beck Anxiety Inventory, Beck Depression Inventory, and SF-36; cost-utility outcome measures were medical direct costs, quality-adjusted life-years (QALYs), and incremental cost-utility ratios (ICURs).

**Results:**

All mCRC patients exhibited a significant decrease in both emotional wellbeing and depression from pretherapeutic period to posttherapeutic 6th month (*P* < 0.05); however, from the posttherapeutic 1st year to the 2nd year, improvement in most QOL measures was significantly better in the study group than in the control group (*P* < 0.05). Over a 2-year time period, the study group had higher total medical direct costs than the control group (US$ 54,742 ± 14,013 vs. US$ 54,608 ± 9,673) and higher average QALYs gained (1.88 vs. 1.65), with an ICUR of US$ 583 per QALY gained.

**Conclusion:**

For patients with mCRC, irinotecan dose escalation appeared cost-effective with considerable QOL improvements during the study period. Further randomized, multi-institutional controlled trials are warranted to corroborate these results.

## Introduction

With the recent advances in pharmacogenomic era, patients undergo genetic testing to determine their genotype before treatment, based on which a suitable medication or dosage is administered (1–3). For metastatic colorectal cancer (mCRC) patients, the standard chemotherapy regimens are 5-fluorouracil (5-FU), leucovorin, and oxaliplatin (FOLFOX); oxaliplatin with capecitabine (CAPOX); or 5-FU, leucovorin, and irinotecan (FOLFIRI). Bevacizumab is a recombinant humanized monoclonal antibody targeting the vascular endothelial growth factor and is usually added to these standard regimens to enhance the efficacy. Our previous multicenter, randomized, controlled, open-label trial study revealed that exceeding the recommended irinotecan dose is safe and effective when a regimen of FOLFIRI plus bevacizumab is administered in mCRC patients with UGT1A1∗1/∗1 and UGT1A1∗1/∗28 genotypes. Additionally, pretherapeutic UGT1A1 genotyping–guided dose adjustment achieved better outcomes compared to the standard regimen. This intention-to-treat (ITT) analysis also demonstrated the benefits of escalating doses of irinotecan under UGT1A1 genotyping. Likewise, other previous studies have also suggested that higher doses of irinotecan in the first-line or later-line setting in mCRC can confer favorable clinical outcomes (4–7).

Roncato et al. estimated the per-patient cost of treating irinotecan-related toxicity associated with the UGT1A1*28 patient genotype. Mean per-patient cost was €812 for UGT1A1*1/*1 patients, €1,119 for UGT1A1*1/*28 patients, and €4,886 for UGT1A1*28/*28 patients (8). Notably, the incremental treatment cost per patient was €4,074 higher in UGT1A1*28/*28 patients compared to UGT1A1*1/*1 patients and €307 higher in UGT1A1*28/*28 patients compared to UGT1A1*1/*28 patients. Kristin et al. also found that the total per-patient cost of a FOLFOX or FOLFIRI chemotherapy regimen was 359 million Indonesian Rupiah (IDR) with a mean average of 1.90 quality-adjusted life-years (QALYs) over a life-time horizon. Additionally, although FOLFOX or FOLFIRI plus bevacizumab increased the total per-patient cost by 108 million IDR, it increased QALYs by 0.17 (9).

Although irinotecan dose escalation demonstrates a more optimal treatment effect in patients with mCRC undergoing *UGT1A1* genotyping and receiving FOLFIRI, the cost utility of different doses of irinotecan plus bevacizumab has not been comprehensively investigated. Additionally, most studies on the economic and cost-effectiveness of this treatment have analyzed the short-term results; however, few studies have analyzed the long-term results. Therefore, the objective of this prospective, long-term follow-up study was to perform a quality of life (QOL) outcome evaluation and a cost-utility analysis of different doses of irinotecan plus bevacizumab in patients with mCRC in the first-line setting.

## Methods

### Study Design and Study Population

**Figure 1:**
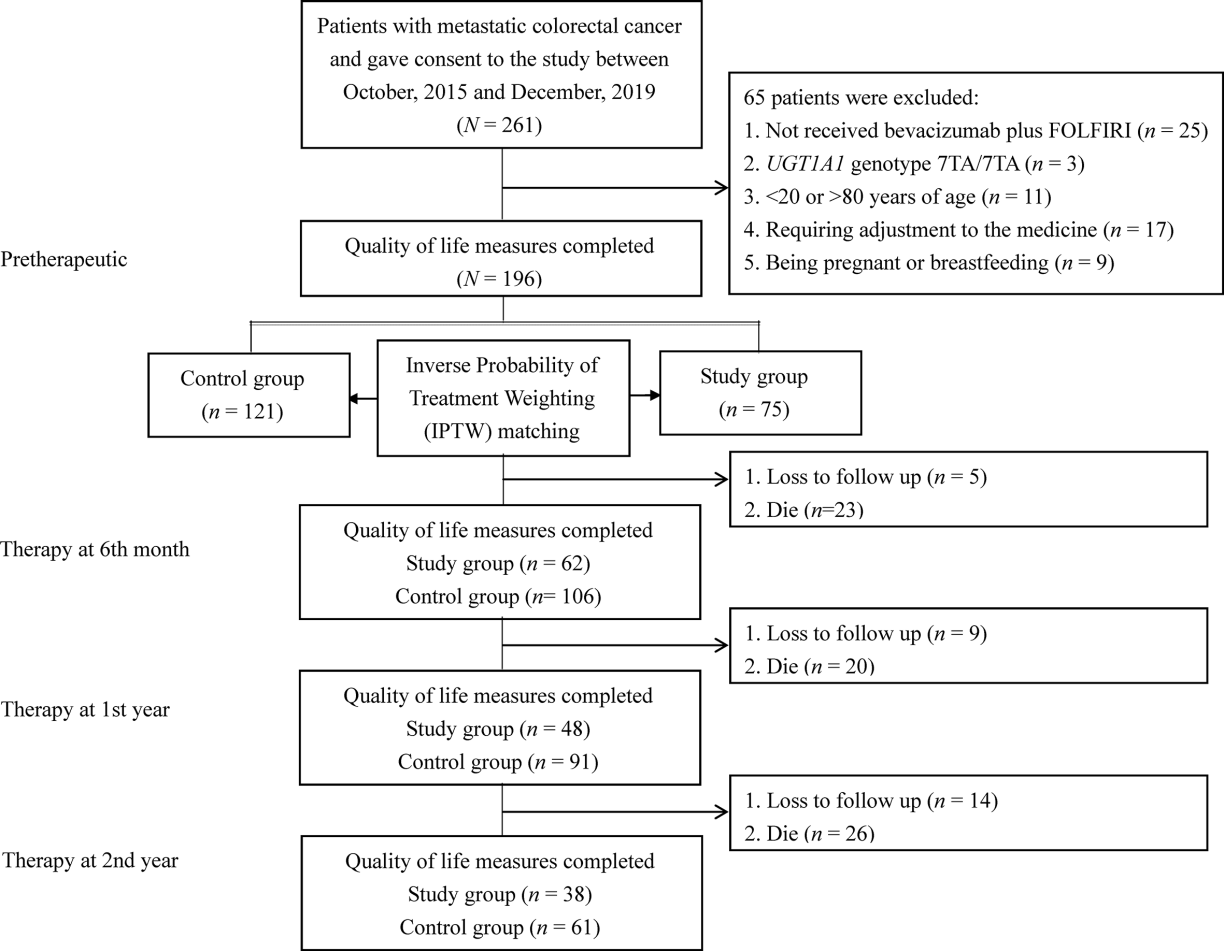
Flowchart of sample selection for prospective cohort analysis.

The study population included patients with mCRC at a medical center between October 2015 and December 2019. The inclusion criteria were as follows: (a) patients who underwent *UGT1A1* genotyping before treatment; (b) mCRC patients with histologically diagnosed adenocarcinoma, and (c) patients aged 20–80 years. The exclusion criteria were as follows: (a) no receipt of bevacizumab plus FOLFIRI, (b) the *UGT1A1* genotype 7TA/7TA (UGT1A1*28*28); (c) <20 or >80 years of age; (d) no dose esclation due to discomfort experienced by those in the dose escalation group; (e) being pregnant or breastfeeding; (f) a major comorbidity; and (g) having not signed the consent form. The recommended irinotecan dose in the FOLFIRI regimen is 180 mg/m^2^ based on a dose-finding study (2, 4, 10). The detailed treatment regimen on irinotecan dose escalation including patient withdrawal was described in our previous study protocol (11). According to the above criteria, mCRC patients were divided into two groups: 75 patients were categorized into the dose escalation group (>180 mg/m^2^, study group) and 121 patients into the recommended dose group (180 mg/m^2^, control group). The patients in the recommended dose group received irinotecan at a dose of 180 mg/m^2^. **Figure 1** shows a flow diagram of the current study illustrating the enrollment, allocation, and matching analysis processes. Before study initiation, approval was obtained from the Institutional Review Board of our hospital (KMUHIRB-(EI)-20150147). The patients/participants provided their written informed consent to participate in this study.

### Quality of Life Measures

The QOL measures included the Functional Assessment of Cancer Therapy-Colorectal (FACT-C) (12), Beck Depression Inventory (BDI) (13), Beck Anxiety Inventory (BAI) (14), and SF-36 (15). Higher FACT-C and SF-36 scores indicated better outcomes, but higher BDI and BAI scores exhibited worse outcomes. All enrolled patients were scheduled to complete all the above assessments at four time points: pretherapeutic and therapy at posttherapeutic 6th month, 1st year, and 2nd year.

### Economic Evaluation

#### Cost Estimation

In accordance with the reimbursement criteria established by the National Health Insurance Administration, medical cost structure included total medical direct costs during therapy and total inpatient costs, total outpatient costs, and total medical emergency costs at posttherapeutic 2 years after discharge. All medical direct costs included expenses for physicians, ward, pharmacy, laboratory, inspection, medical materials, etc. All cost inputs were adjusted to US$ 2019 according to the consumer price index (CPI) and discounted annually by 3%.

#### Estimation of Utility

To estimate QALYs, cost-utility analysis often uses “utility scores” (health state valuations) anchored by 0 and 1, where 0 indicates death and 1 indicates full health. This study used the time trade-off valuation procedure to convert EuroQol-5D (EQ-5D) total scores to utility scores (16). The utilities reported by each patient were multiplied by the assumed duration of sustained benefit after intervention (summed up to the end of 2 years) to estimate QALYs. To maintain consistency with QALYs calculation, this study assumed that the only resources used by patients were those captured during the 2 years of follow-up. That is, the analysis assumed that patients did not incur any other healthcare costs during the remainder of the year.

### Statistical Analysis

In the main patient-level analysis, we used inverse probability-of-treatment weighting (IPTW) based on propensity score to construct a weighted cohort of patients with different doses of irinotecan plus bevacizumab but similar with respect to other study characteristics. IPTW based on propensity scores was used to account for differences in study characteristics between the two regimens (17). The resulting differences between the weighted groups of interest reflected the average treatment effect (**Table 1**). The distribution of FACT-C scores at each time point was analyzed in terms of median, range and interquartile range and visualized by box-and-whisker plots.

**Table 1:** Study characteristics before and after IPTW^*^.

Variables		Total (*N*=196)	Before IPTW matching			After IPTW matching		
			Study group (*n*=75)	Control group (*n*=121)	*P* value	Study group (*n*=75)	Control group (*n*=121)	*P* value
Gender	Male	125 (63.8)	54 (72.0)	71 (58.7)	0.083^†^	65.3	65.0	1.000^†^
	Female	71 (36.2)	21 (28.0)	50 (41.3)		34.7	35.0	
Age, years		58.74 ± 12.06	56.11 ± 13.21	60.74 ± 10.77	0.021^#^	59.13 ± 12.08	59.98 ± 10.80	0.897^#^
Education, years		10.67 ± 3.68	11.22 ± 3.04	10.34 ± 3.40	0.084^#^	10.66 ± 3.22	10.66 ± 3.83	0.993^#^
Body mass index, kg/m^2^		22.95 ± 3.45	22.37 ± 3.43	23.30 ± 3.43	0.070^#^	22.57 ± 3.26	22.79 ± 3.52	0.534^#^
Marital status	Married	158 (80.6)	63 (84.0)	95 (78.5)	0.448^†^	87.6	85.3	0.665^†^
	Unmarried	38 (19.4)	12 (16.0)	26 (21.5)		12.4	14.7	
Smoking	Yes	36 (18.4)	18 (24.0)	18 (14.9)	0.157^†^	19.7	19.2	1.000^†^
	No	160 (81.6)	57 (76.0)	103 (85.1)		80.3	80.8	
Drinking	Yes	50 (25.5)	21 (28.0)	29 (24.0)	0.645^†^	25.4	25.3	1.000^†^
	No	146 (74.5)	54 (72.0)	92 (76.0)		74.6	74.7	
Die	Yes	69 (35.2)	35 (46.7)	34 (28.1)	0.013^†^	34.7	36.4	0.814^†^
	No	127 (64.8)	40 (53.3)	87 (71.9)		65.3	63.6	

IPTW, inverse probability of treatment weighting.

^*^The values are presented as mean ± standard deviation or n (%).

^†^P-values calculated by Student’s t-test.

^#^P-values calculated by chi-square test.

A common limitation of longitudinal studies is the absence of an appropriate statistical methodology that can control for censoring and intercorrelations that occur when measures are repeatedly obtained for the same pool of subjects. To address this limitation, this study used a generalized estimating equation (GEE) model to cluster mCRC patients; the GEE model was also useful for generating propensity scores. Moreover, total scores for each QOL measure were compared between the study group and control group by using the differences-in-differences (DID) methodology (i.e., a pre–post study design with a comparison group) (18). Standard errors in DID in the predicted values were estimated using the bootstrap technique (1,000 replications and sample sizes equal to the original sample size) (19). Additionally, effect size was obtained using Cohen’s d statistics (i.e., the difference between the mean posttherapeutic QOL value and the mean pretherapeutic QOL value divided by the pooled standard deviation) (20).

After converting EQ-5D scores into utility scores, the number of QALYs over a period of 2 years was calculated for each patient by using the area under the curve approach after controlling for imbalances in baseline utility scores (21). The incremental cost-utility ratio (ICUR) was calculated as the ratio of the difference in mean cost per patient to the difference in mean QALYs per patient between the study and control groups. A willingness-to-pay (WTP) threshold of gross domestic product (GDP) US$ 26,263.5 per QALY was used to assess cost-effectiveness. A regimen is termed dominant when it is both less costly and more effective. To derive cost-effectiveness acceptability curve, this study performed nonparametric bootstrapping on the incremental cost and effectiveness with 1,000 replications and a cost-effectiveness acceptability frontier. All *P* values reported were two-sided, and *P* values of <0.05 were considered statistically significant. The software package used to perform GEE in all statistical analyses was xtgee in Stata version 13.0 (Stata Corp LP, College Station, TX, USA), and a decision tree model was built using the TreeAge Pro 2017 (Tree-Age Software Inc. Williamstown, MA, USA).

## Results

To unify the study characteristics of the two groups and reinforce the subsequent research results, IPTW was used to match all study characteristics; thus, no variables were significantly different between the groups (**Table 1**).

### Changing Trends of QOL in the Two Groups After IPTW

**Figure 2:**
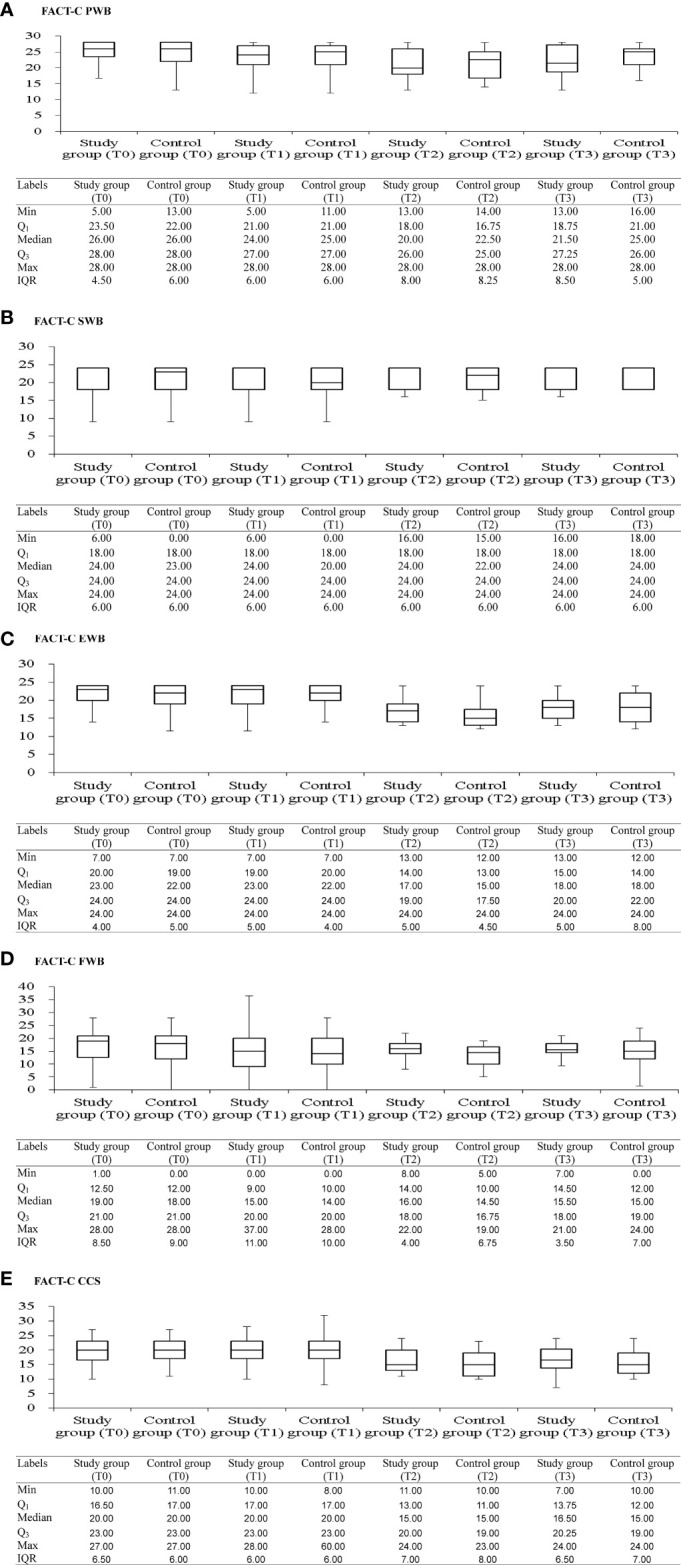
Box and whisker plots of the FACT-C scores [**(A)** FACT-C PWB; **(B)** FACT-C SWB; **(C)** FACT-C EWB; **(D)** FACT-C EWB; **(E)** FACT-C CCS] at each time-point, with study and control groups presented side by side. The box contains 50% of all values (the 25th to 75th percentile) and is divided by the horizontal bar which is the median value (50th percentile). FACT-C, functional assessment of cancer therapy-colorectal; PWB, physical wellbeing; SWB, social/family wellbeing; EWB, emotional wellbeing; FWB, functional wellbeing; CCS, colorectal cancer specific wellbeing; T0, pretherapeutic; T1, posttherapeutic 6th month; T2, posttherapeutic 1st year; T3, posttherapeutic 2nd year.

**Figure 2** shows box plots illustrating the FACT-C score distributions in the study and control groups at different time points. From pretherapeutic to posttherapeutic 6th month after discharge, mCRC patients exhibited a significant decrease in FACT-C functional wellbeing (ES = −1.05 in the study group and ES = −0.35 in the control group), SF-36 physical functioning (ES = −0.39 in the study group and ES = −0.37 in the control group), and depression function (ES = 0.28 in the study group and ES = 0.13 in the control group; **Tables 2**, **3**). At the remaining follow-up time points, different changing trends in QOL values in the two groups were noted.

**Table 2:** Paired t test was employed to evaluate trends of the FACT-C, BDI, BAI, and SF-36 in the study group in patients with metastatic colorectal cancer at different time points (*N*=75).

Measures	Pretherapeutic (T_0_)	Posttherapeutic 6th month (T_1_)			Posttherapeutic 1st year (T_2_)			Posttherapeutic 2nd year (T_3_)		
	Mean± SD	Mean± SD	ES^†^	*P* value^¶^	Mean± SD	ES^§^	*P* value^¶^	Mean± SD	ES^#^	*P* value^¶^
FACT-C PWB	24.83 ± 4.27	22.28 ± 5.10	-0.60	0.004	23.47 ± 4.68	0.23	<0.001	24.62 ± 4.91	0.25	<0.001
FACT-C SWB	21.59 ± 3.96	21.37 ± 3.99	-0.06	0.604	22.20 ± 2.94	0.21	0.777	21.75 ± 3.05	-0.15	0.808
FACT-C EWB	21.43 ± 3.70	17.55 ± 3.35	-1.05	<0.001	21.10 ± 3.75	1.06	<0.001	18.76 ± 3.12	0.36	<0.001
FACT-C FWB	17.50 ± 6.17	15.47 ± 7.06	-0.33	0.008	15.02 ± 3.19	-0.06	0.480	15.64 ± 3.53	0.19	0.640
FACT-C CCS	19.96 ± 4.08	19.80 ± 4.07	-0.04	0.692	16.48 ± 4.07	-0.82	<0.001	18.30 ± 5.07	0.45	0.020
SF36 PF	87.01 ± 17.32	80.20 ± 20.98	-0.39	0.001	83.41 ± 6.53	0.15	0.392	93.08 ± 8.95	1.48	<0.001
SF36 RP	59.59 ± 49.94	44.92 ± 48.65	-0.29	<0.001	56.33 ± 45.99	0.23	0.044	62.09 ± 47.31	0.13	0.027
SF36 BP	78.54 ± 23.93	80.25 ± 27.49	0.07	0.021	86.25 ± 18.33	0.25	0.024	87.14 ± 18.80	0.05	0.085
SF36 GH	56.02 ± 21.13	54.01 ± 20.78	-0.10	0.450	59.26 ± 18.56	0.18	0.081	66.06 ± 22.48	0.37	<0.001
SF36 VT	66.65 ± 19.73	64.19 ± 19.39	-0.12	0.273	65.38 ± 20.44	0.06	0.133	68.79 ± 19.84	0.17	0.193
SF36 SF	70.73 ± 25.36	68.82 ± 30.49	-0.08	0.629	73.23 ± 22.86	0.14	0.497	74.33 ± 21.20	0.05	0.697
SF36 RE	72.06 ± 43.97	73.50 ± 43.21	0.03	0.813	75.91 ± 32.63	0.06	0.105	85.75 ± 32.82	0.30	0.532
SF36 MH	72.65 ± 19.03	71.59 ± 20.01	-0.06	0.613	72.35 ± 15.86	0.04	0.939	76.04 ± 15.25	0.23	0.038
BDI	2.88 ± 4.23	4.05 ± 6.65	0.28	0.189	0.70 ± 2.25	-0.50	<0.001	0.41 ± 1.38	-0.13	0.298
BAI	0.11 ± 0.77	0.07 ± 0.42	-0.05	0.677	0.05 ± 0.37	-0.05	0.749	0.01 ± 0.52	-0.11	0.055

FACT-C, functional assessment of cancer therapy-colorectal; PWB, physical wellbeing; SWB, social/family wellbeing; EWB, emotional wellbeing; FWB, functional wellbeing; CCS, colorectal cancer specific wellbeing; PF, physical functioning; RP, role limitation due to physical problems; BP, bodily pain; GH, general health; VT, vitality; SF, social functioning; RE, role limitation due to emotional problems; MH, mental health; BDI, Beck depression inventory; BAI, Beck anxiety inventory; SD, standard deviation; ES, effect size.

^†^T_1_ vs. T_0_, ^§^T_2_ vs. T_1_, ^#^T_3_ vs. T_2_.

^¶^P-values calculated by paired t-test.

**Table 3:** Paired t test was employed to evaluate trends of the FACT-C, BDI, BAI, and SF-36 in the control group in patients with metastatic colorectal cancer at different time points (*n*=121).

Measures	Pretherapeutic (T_0_)	Posttherapeutic 6th month (T_1_)			Posttherapeutic 1st year (T_2_)			Posttherapeutic 2nd year (T_3_)		
	Mean± SD	Mean± SD	ES^†^	*P* value^¶^	Mean± SD	ES^§^	*P* value^¶^	Mean± SD	ES^#^	*P* value^¶^
FACT-C PWB	24.60 ± 3.48	22.70 ± 4.25	-0.55	0.013	21.54 ± 4.69	-0.27	0.302	23.22 ± 3.88	0.36	0.758
FACT-C SWB	20.70 ± 3.78	20.73 ± 3.72	0.01	0.926	21.22 ± 3.27	0.13	0.933	21.29 ± 2.98	0.26	0.767
FACT-C EWB	20.84 ± 3.95	21.04 ± 3.80	0.05	0.381	16.18 ± 4.10	-1.28	<0.001	17.94 ± 4.27	0.43	0.088
FACT-C FWB	16.44 ± 7.29	13.87 ± 6.97	-0.35	<0.001	13.40 ± 4.61	-0.07	0.252	14.91 ± 5.53	0.33	0.387
FACT-C CCS	19.44 ± 3.81	19.42 ± 5.95	-0.01	0.979	15.20 ± 4.63	-0.71	<0.001	15.76 ± 4.15	0.12	0.562
SF36 PF	83.94 ± 27.61	73.59 ± 27.77	-0.37	<0.001	80.78 ± 13.17	0.26	0.075	82.83 ± 19.26	0.16	0.905
SF36 RP	53.95 ± 48.56	42.63 ± 48.75	-0.23	0.004	47.10 ± 53.92	0.09	0.724	49.79 ± 51.89	0.05	0.609
SF36 BP	79.51 ± 20.70	76.95 ± 24.18	-0.12	0.244	80.96 ± 22.97	0.17	0.508	69.87 ± 22.34	-0.48	0.121
SF36 GH	58.45 ± 19.63	55.32 ± 21.21	-0.16	0.034	48.23 ± 10.43	-0.33	0.759	64.10 ± 24.01	1.52	0.006
SF36 VT	63.43 ± 22.84	59.42 ± 23.92	-0.18	0.023	55.53 ± 29.05	-0.16	0.421	61.88 ± 17.76	0.22	0.124
SF36 SF	75.07 ± 23.32	68.85 ± 34.66	-0.27	0.049	72.04 ± 24.45	0.09	0.525	68.30 ± 22.51	-0.15	0.318
SF36 RE	72.13. ± 23.32	63.63 ± 48.10	-0.36	0.066	72.04 ± 24.45	0.37	0.014	74.94 ± 44.97	0.12	0.074
SF36 MH	73.17 ± 16.47	70.98 ± 17.95	-0.13	0.150	75.55 ± 12.35	0.25	0.862	67.22 ± 14.54	-0.67	0.038
BDI	3.85 ± 5.81	4.60 ± 6.68	0.13	0.264	0.39 ± 1.75	-0.63	<0.001	0.26 ± 1.26	-0.07	0.554
BAI	0.61 ± 3.3	0.81 ± 3.4	0.06	0.045	0.01 ± 0.09	-0.24	0.002	0.01 ± 0.10	0.01	0.885

FACT-C, functional assessment of cancer therapy-colorectal; PWB, physical wellbeing; SWB, social/family wellbeing; EWB, emotional wellbeing; FWB, functional wellbeing; CCS, colorectal cancer specific wellbeing; PF, physical functioning; RP, role limitation due to physical problems; BP, bodily pain; GH, general health; VT, vitality; SF, social functioning; RE, role limitation due to emotional problems; MH, mental health; BDI, Beck depression inventory; BAI, Beck anxiety inventory; SD, standard deviation; ES, effect size.

^†^T_1_ vs. T_0_, ^§^T_2_ vs. T_1_, ^#^T_3_ vs. T_2_.

^¶^P-values calculated by paired t-test.

### Differences in the QOL Between the Two Groups After IPTW

**Table 4** presents a comparison of the differences in QOL measures between the two groups at four time points: pretherapeutic (T0), posttherapeutic 6th month (T1), posttherapeutic 1st year (T2), and posttherapeutic 2nd year (T3). Compared with the control group, at T0, the study group had a significantly better function in FACT-C social/family wellbeing (*P* = 0.024) and BAI (*P* = 0.039); at T1, the study group had a significantly better function in FACT-C functional wellbeing (*P* = 0.025), BAI (*P* = 0.003), SF-36 physical functioning (*P* = 0.008), vitality (*P* = 0.031), and role limitation due to emotional problems (*P* = 0.033); at T2, the study group had a significantly better function in FACT-C physical wellbeing (*P* = 0.025), FACT-C emotional wellbeing (*P* = 0.002), and SF-36 role limitation due to physical problems (*P* = 0.033); at T3, the study group had a significantly better function in FACT-C colorectal cancer-specific wellbeing (*P* = 0.017), BAI (*P* = 0.009), SF-36 physical functioning (*P* = 0.002), role limitation due to physical problems (*P* = 0.004), and role limitation due to emotional problems (*P* = 0.017). Overall, from posttherapeutic 1st year to the 2nd year, improvements in most QOL measures were significant in the study group compared with those in the control group (*P* < 0.05).

**Table 4:** Generalized estimating equations (GEE) model with difference-in-difference (DID) method was employed to evaluate differences in the FACT-C, BDI, BAI, and SF-36 between the two groups in patients with metastatic colorectal cancer at different time points (*N*=196).

Measures		Pretherapeutic		Posttherapeutic 6th month		Posttherapeutic 1st year		Posttherapeutic 2nd year	
		Mean ± SE	*P* value^¶^	Mean ± SE	*P* value^¶^	Mean ± SE	*P* value^¶^	Mean ± SE	*P* value^¶^
FACT-C PWB	Study group	24.83 ± 0.31	0.567	22.28 ± 0.37	0.370	23.47 ± 0.68	0.025	24.62 ± 0.64	0.786
	Control group	24.60 ± 0.25		22.70 ± 0.30		21.54 ± 1.04		23.22 ± 0.75	
	Differences	0.23 ± 0.39		-0.42 ± 0.48		1.93 ± 1.25		1.40 ± 0.98	
FACT-C SWB	Study group	21.59 ± 0.29	0.024	21.37 ± 0.29	0.104	22.20 ± 0.42	0.235	21.75 ± 0.40	0.519
	Control group	20.70 ± 0.27		20.73 ± 0.26		21.22 ± 0.73		21.29 ± 0.57	
	Differences	0.89 ± 0.39		0.64 ± 0.39		0.98 ± 0.81		0.46 ± 0.71	
FACT-C EWB	Study group	21.43 ± 0.27	0.127	17.55 ± 0.27	0.858	21.10 ± 0.48	0.002	18.76 ± 0.41	0.377
	Control group	21.04 ± 0.27		20.84 ± 0.28		16.18 ± 0.92		17.94 ± 0.82	
	Differences	0.39 ± 0.39		-3.29 ± 0.38		4.92 ± 0.96		0.82 ± 0.91	
FACT-C FWB	Study group	17.50 ± 0.44	0.119	15.47 ± 0.51	0.025	15.02 ± 0.46	0.161	15.64 ± 0.46	0.532
	Control group	16.44 ± 0.52		13.87 ± 0.50		13.40 ± 1.03		14.91 ± 1.06	
	Differences	1.06 ± 0.68		1.59 ± 0.71		1.63 ± 1.13		0.73 ± 1.16	
FACT-C CCS	Study group	19.96 ± 0.29	0.189	19.80 ± 0.29	0.469	16.48 ± 0.59	0.263	18.30 ± 0.66	0.017
	Control group	19.44 ± 0.27		19.42 ± 0.42		15.20 ± 1.04		15.76 ± 0.80	
	Differences	0.53 ± 0.40		0.37 ± 0.52		1.28 ± 1.14		2.54 ± 1.03	
BDI	Study group	2.88 ± 0.30	0.060	4.05 ± 0.48	0.411	0.70 ± 0.17	0.154	0.41 ± 0.10	0.332
	Control group	3.85 ± 0.41		4.60 ± 0.47		0.39 ± 0.14		0.26 ± 0.10	
	Differences	-0.97 ± 0.51		-0.55 ± 0.67		0.32 ± 0.22		0.14 ± 0.15	
BAI	Study group	0.11 ± 0.77	0.039	0.07 ± 0.03	0.003	0.05 ± 0.03	0.110	0.01 ± 0.04	0.009
	Control group	0.61 ± 0.24		0.81 ± 0.24		0.01 ± 0.01		0.11 ± 0.01	
	Differences	-0.50 ± 0.24		-0.74 ± 0.25		0.05 ± 0.03		-0.10 ± 0.04	
**SF36**		**Pretherapeutic**		**Posttherapeutic 6th month**		**Posttherapeutic 1st year**		**Posttherapeutic 2nd year**	
		**Mean ± SE**	***P* value^¶^**	**Mean ± SE**	***P* value^¶^**	**Mean ± SE**	***P* value^¶^**	**Mean ± SE**	***P* value^¶^**
PF	Study group	87.01 ± 1.25	0.187	80.20 ± 1.51	0.008	83.41 ± 1.21	0.518	93.08 ± 1.19	0.002
	Control group	83.94 ± 1.96		73.59 ± 1.97		80.78 ± 3.77		82.83 ± 4.08	
	Differences	3.07 ± 2.32		6.60 ± 2.48		2.63 ± 3.96		10.25 ± 3.16	
RP	Study group	59.59 ± 3.59	0.258	44.92 ± 3.50	0.641	56.33 ± 8.54	0.033	62.09 ± 6.29	0.004
	Control group	53.95 ± 3.45		42.63 ± 3.46		47.10 ± 15.4		49.79 ± 10.98	
	Differences	5.64 ± 4.98		2.29 ± 4.92		9.23 ± 16.67		12.30 ± 12.22	
BP	Study group	78.54 ± 1.72	0.668	80.25 ± 1.98	0.208	86.25 ± 3.40	0.441	77.14 ± 2.50	0.149
	Control group	79.51 ± 1.47		76.95 ± 1.72		80.96 ± 6.58		69.87 ± 4.73	
	Differences	-0.97 ± 0.27		3.30 ± 2.61		5.29 ± 6.80		7.27 ± 4.98	
GH	Study group	56.02 ± 1.52	0.238	54.01 ± 1.49	0.535	39.26 ± 3.44	0.057	66.06 ± 2.99	0.735
	Control group	58.45 ± 1.39		55.32 ± 1.51		48.23 ± 2.99		64.10 ± 5.08	
	Differences	-2.43 ± 2.06		-1.32 ± 2.12		-8.97 ± 4.56		1.96 ± 5.76	
VT	Study group	66.65 ± 1.42	0.136	64.19 ± 1.39	0.031	55.38 ± 3.80	0.975	63.79 ± 2.64	0.695
	Control group	63.43 ± 1.62		59.42 ± 1.70		55.63 ± 8.32		61.88 ± 3.76	
	Differences	3.22 ± 2.16		4.77 ± 2.20		-0.25 ± 7.95		1.91 ± 4.86	
SF	Study group	70.73 ± 1.82	0.079	68.82 ± 2.19	0.991	73.23 ± 4.24	0.884	72.33 ± 2.82	0.459
	Control group	75.07 ± 1.66		68.85 ± 2.46		72.04 ± 7.00		68.30 ± 4.77	
	Differences	-4.33 ± 2.46		-0.04 ± 3.20		1.19 ± 8.06		4.04 ± 5.42	
RE	Study group	72.06 ± 3.16	0.987	73.50 ± 3.11	0.033	85.92 ± 6.06	0.685	85.75 ± 4.36	0.017
	Control group	72.13 ± 3.20		63.63 ± 3.42		90.49 ± 90.7		74.96 ± 9.52	
	Differences	-0.08 ± 4.50		9.87 ± 4.62		-4.58 ± 11.21		10.79 ± 10.47	
MH	Study group	72.62 ± 1.37	0.775	71.59 ± 1.44	0.753	72.35 ± 2.95	0.543	70.04 ± 2.03	0.460
	Control group	73.17 ± 1.17		70.98 ± 1.27		75.55 ± 3.54		67.22 ± 3.08	
	Differences	-0.52 ± 1.80		0.60 ± 1.92		-3.20 ± 5.20		2.81 ± 3.69	

FACT-C, functional assessment of cancer therapy-colorectal; PWB, physical wellbeing; SWB, social/family wellbeing; EWB, emotional wellbeing; FWB, functional wellbeing; CCS, colorectal cancer specific wellbeing; BDI, Beck depression inventory; BAI, Beck anxiety inventory; SE, standard error; T_0_, pre-therapeutic, T_1_, post-therapeutic 6^th^ month; T_2_, post-therapeutic 1^st^ year; T_3_, post-therapeutic 2^nd^ year.

^¶^P-values calculated by GEE model.

### Cost-Utility Analysis of the Study Group Compared With the Control Group

**Figure 3:**
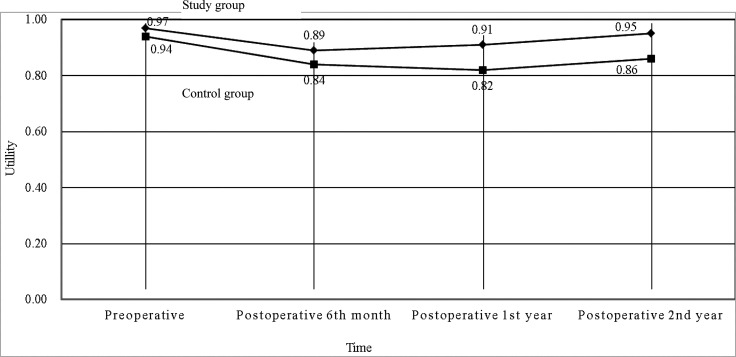
Utilities of the two groups in patients with metastatic colorectal cancer at different time points (*N* = 196).

The mean total medical direct costs per patient over a 2-year time period included the mean total medical direct costs during therapy and the mean total medical direct costs at posttherapeutic 2 years after discharge. The mean total medical direct cost of the study group was US$ 54,742 (standard deviation, SD, US$ 14,013) compared with US$ 54,608 (SD US$ 9,673) for the control group, resulting in mean incremental costs of US$ 134 (**Table 5**). Treatment in the study group led to a decrease in the mean utility score from 0.97 (SD 0.09) at the pretherapeutic time point to 0.95 (SD 0.09) at posttherapeutic 2nd year compared with the control group, which had a mean of 0.94 (SD 0.16) at the pretherapeutic time point to 0.86 (SD 0.24) at posttherapeutic 2nd year (**Table 6** and **Figure 3**). After adjusting for pretherapeutic utility, the mean QALYs per patient in the study group was 1.88, whereas the mean QALYs per patient in the control group was 1.65. This resulted in an ICUR of US$ 583 per QALY over the first 2 years for patients in the study group.

**Table 5:** Comparisons of means and standard deviations of medical direct costs between the two groups in patients with metastatic colorectal.

Items	Total (*N*=196)	Study group (*n*=75)	Control group (*n*=121)	*P* value^¶^
Total medical direct costs during therapy	2,289 ± 747	2,330 ± 896	2,250 ± 567	0.035
Total numbers of inpatient during the study period	17.9 ± 8.8	17.7 ± 9.2	18.1 ± 8.4	0.355
Posttherapeutic 6 months after discharge				
Total inpatient costs	17,543 ± 5,620	18,493 ± 5,965	16,614 ± 5,096	<0.001
Total outpatient costs	1,304 ± 1,742	1,119 ± 1,701	1,485 ± 1,763	<0.001
Health insurance reimbursement expenses	1,105 ± 1,336	891 ± 1,162	1,314 ± 1,461	<0.001
Self-paid (out of pocket) expenses	199 ± 628	227 ± 714	171 ± 532	0.078
Total medical emergency costs	59 ± 136	58 ± 162	60 ± 103	0.235
Health insurance reimbursement expenses	57 ± 129	57 ± 154	58 ± 99	0.855
Self-paid (out of pocket) expenses	2 ± 1	2 ± 12	2 ± 8	0.411
Posttherapeutic 1 year after discharge				
Total inpatient costs	31,014 ± 7,501	32,360 ± 8,304	29,697 ± 6,391	<0.001
Total outpatient costs	2,164 ± 2,338	1,998 ± 2,044	2,326 ± 2,592	0.006
Health insurance reimbursement expenses	1,879 ± 2,123	1,648 ± 1,620	2,106 ± 2507	<0.001
Self-paid (out of pocket) expenses	285 ± 698	350 ± 793	220 ± 586	<0.001
Total medical emergency costs	125 ± 235	97 ± 174	153 ± 281	<0.001
Health insurance reimbursement expenses	124 ± 231	95 ± 166	151 ± 279	<0.001
Self-paid (out of pocket) expenses	2 ± 10	2 ± 12	2 ± 6	0.715
Posttherapeutic 2 years after discharge				
Total inpatient costs	49,161 ± 11,522	48,859 ± 13,459	49,456 ± 9,242	0.308
Total outpatient costs	3,085 ± 3,562	3,425 ± 4,402	2,753 ± 2,438	<0.001
Health insurance reimbursement expenses	2,819 ± 3,221	3,051 ± 3,902	2,592 ± 2,371	0.005
Self-paid (out of pocket) expenses	266 ± 680	374 ± 889	160 ± 351	<0.001
Total medical emergency costs	138 ± 232	128 ± 227	149 ± 236	0.066
Health insurance reimbursement expenses	135 ± 228	124 ± 223	146 ± 232	0.057
Self-paid (out of pocket) expenses	3 ± 19	4 ± 26	3 ± 9	0.731

cancer at different time point.

^¶^P-values calculated by Student’s t-test.

**Table 6:** Cost utility analysis of the study group compared to the control group in patients with metastatic colorectal cancer over a 2-year time horizon (*N*=196).

Items	Study group (*n*=75)	Control group (*n*=121)
Total medical direct costs	54,742 ± 14,013	54,608 ± 9,673
Incremental costs	134	
Utility		
Pretherapeutic	0.97 ± 0.09	0.94 ± 0.16
Posttherapeutic 6^th^ month	0.89 ± 0.20	0.84 ± 0.25
Posttherapeutic 1^st^ year	0.91 ± 0.14	0.82 ± 0.29
Posttherapeutic 2^nd^ year	0.95 ± 0.09	0.86 ± 0.24
Quality adjusted life years (QALYs)	1.88	1.65
Incremental QALYs	0.23	
Incremental cost utility ratio (ICUR)	583	

### Sensitivity Analysis

#### Probabilistic Sensitivity Analysis (PSA)

**Figure 4:**
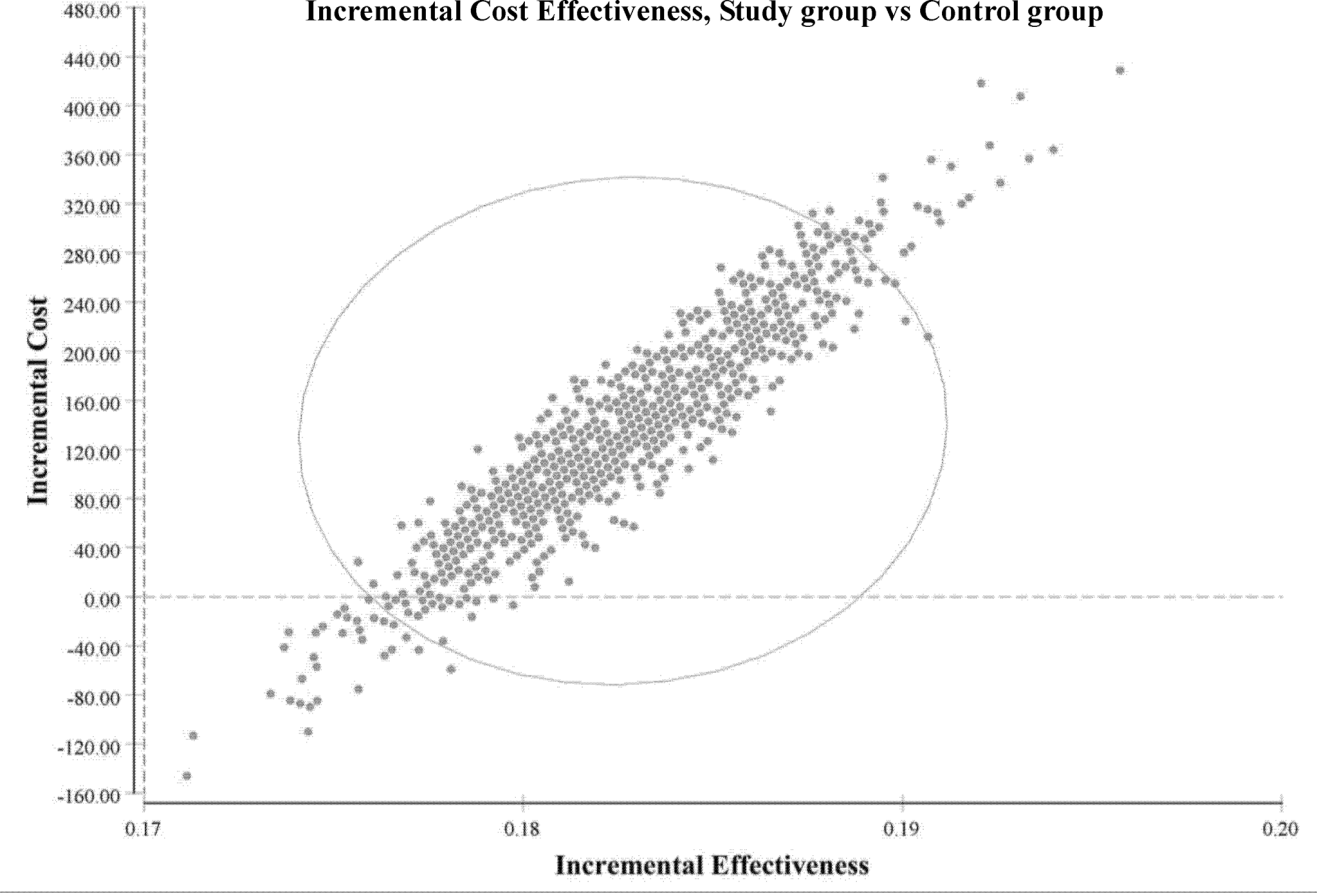
Incremental cost-effectiveness (study group vs. control group). Scatter plots of incremental effectiveness (quality-adjusted life-years) versus incremental costs from 1,000 resamplings in the probabilistic sensitivity analysis with variation limited to cost and effectiveness assumptions and with transition-probabilities constant. This probabilistic sensitivity analysis demonstrated that the study group had a 100% probability of achieving cost-effectiveness relative to the control group in patients with metastatic colorectal cancer. Each plotted point is the result of an incremental cost divided by incremental quality-adjusted life-years. The elliptic circle represents the 95% confidence interval.

**Figure 5:**
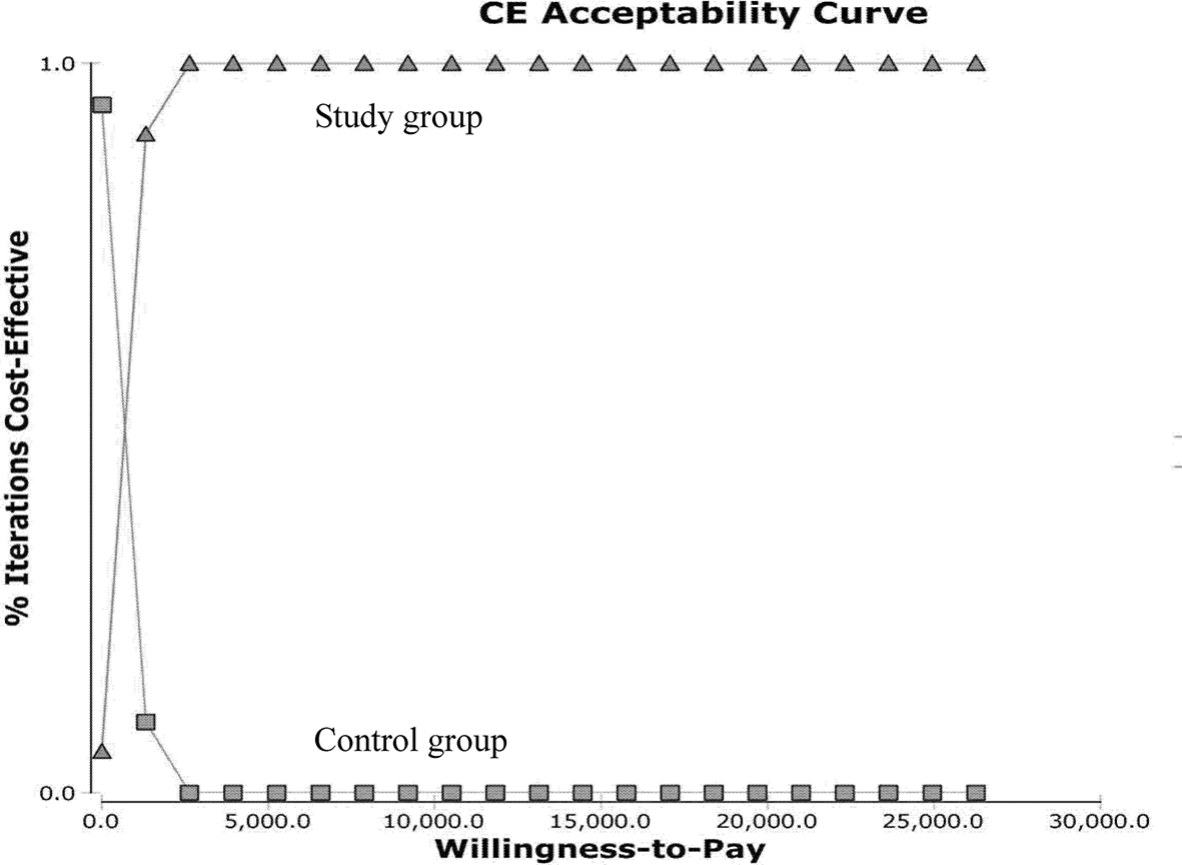
Cost-effectiveness acceptability curves for the probabilistic sensitivity analysis. Results from 1,000 resamplings in the probabilistic sensitivity analysis created synthetic populations of patients from the trial using bootstrapping. The lines represent the fraction of simulation iterations in which the study group achieved more cost-effectiveness than the control group (y-axis) at various levels of willingness to pay for quality-adjusted life-year (QALYs) gains (x-axis).

The ICURs for the 1,000 samples in the PSA are shown in the scatter plot (**Figure 4**). All points were under the US$ 26,263.5-per-QALY level, and 100% of the tested ICURs were in the northeastern quadrant. The cost-effectiveness acceptability curve is also shown in **Figure 5** for varying values of WTP per QALY.

#### Univariable Sensitivity Analysis (USA)

**Figure 6:**
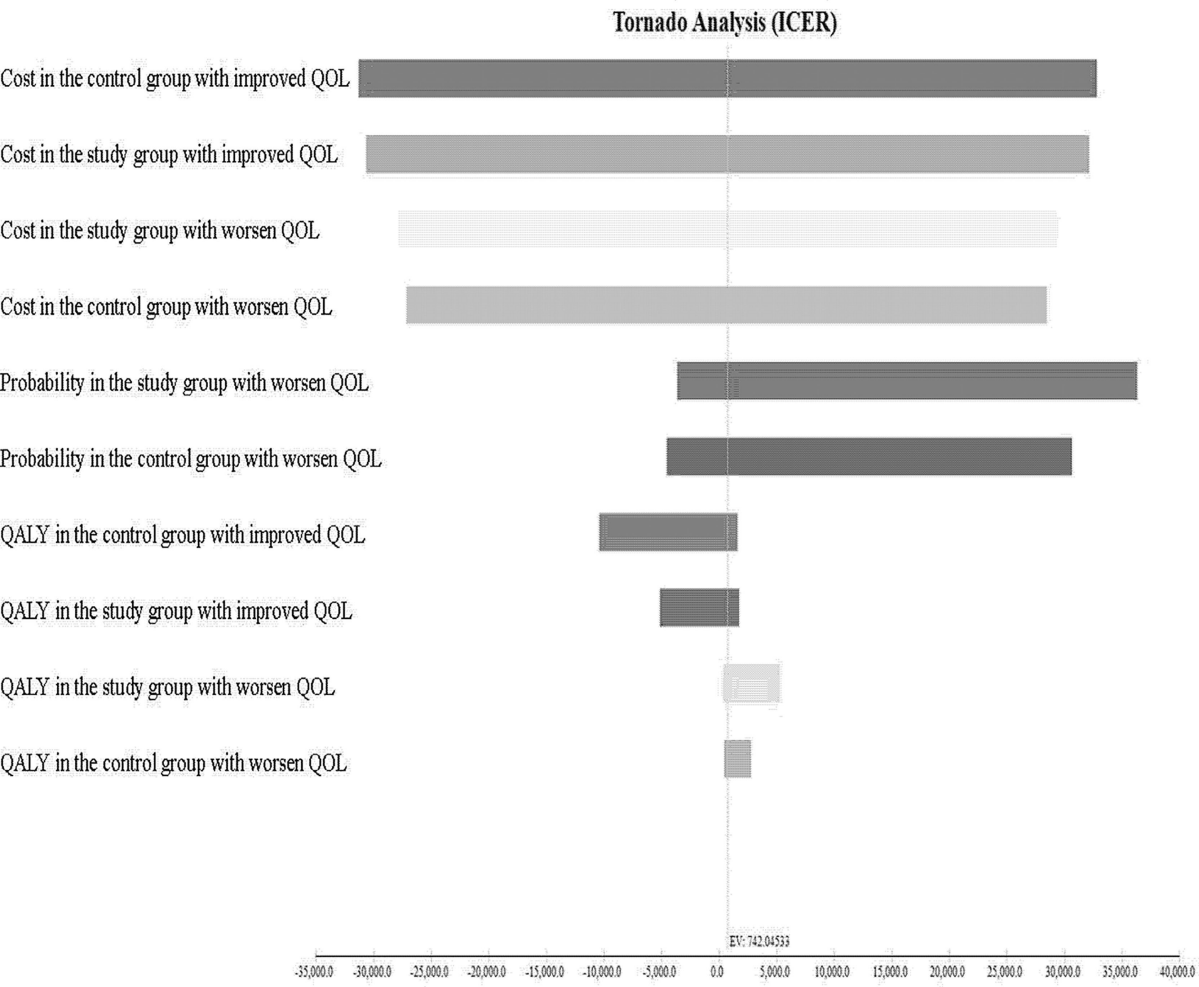
Tornado diagram showing one-way sensitivity analysis results. Costs are expressed in US$ 2020. ICUR, incremental cost-utility ratio; QOL, quality of life.

The results of the USA are shown in the tornado diagram (**Figure 6**). The parameters with the greatest influence on the ICUR were total costs in the study group with improved QOL, followed by total costs in the study group with maintained QOL, and total costs in the control group with maintained QOL. Even with a broad variation in range for each parameter, the ICUR remained below US$ 26,263.5 per QALY.

## Discussion

In both groups of patients with mCRC, physiological functions and emotional wellbeing significantly worsened at posttherapeutic 6th month compared with the pretherapeutic status. This can probably be attributed to the adverse effect of the chemotherapy. The study group exhibited significant improvement in physiological functions and emotional wellbeing from posttherapeutic 6th month to posttherapeutic 1st year. However, the control group exhibited worse emotional wellbeing, possibly because their physiological functions had not fully recovered. From posttherapeutic 1st year to posttherapeutic 2nd year, the study group gradually improved in most QOL measures. By contrast, the control group exhibited slow improvement in physiological functions, and their moods were still affected. These results were consistent with previous reports that compared patient conditions before chemotherapy and radiotherapy; the physiological functions, social functions, and nausea and vomit symptoms of cancer patients worsened during the 6-month treatment, leading to negative emotional functions (22–24). However, most QOL outcomes improved but they still were inferior to the initial status at 1–2 years after chemotherapy and radiotherapy. These results emphasized that baseline characterization of symptom status should be incorporated into clinical trials as they may affect symptom burden.

Compared with the control group, the study group had higher mean total medical direct costs, possibly because of the dose difference. The study group had higher hospitalization expenses during therapy and subsequent hospitalization expenses compared with the control group. From pretherapeutic to posttherapeutic 6th month, patients with mCRC in both groups had significantly reduced utility, possibly because they both initially received accumulated doses of irinotecan and had substantial adverse effects. However, at posttherapeutic 1st year, the study group exhibited gradual recovery and a significant increase in their utility, whereas the control group showed a significant decline from posttherapeutic 6th month to posttherapeutic 1st year, only showing a gradual increase at the posttherapeutic 2nd year. This may be because the study group patients undergo *UGT1A1* genotyping before treatment, and better oncological outcomes were verified by our previous study (2). In addition, Shulman et al. and Lu et al. revealed that in treating mCRC using FOLFIRI combined with bevacizumab, patients with genotypes UGT1A1*28/*28 had significantly increased grades III or IV hematological toxicity than those with UGT1A1*1/*28 and UGT1A1*1/*1 (10, 25). Gradual irinotecan dose escalation according to *UGT1A1* genotypes can yield better treatment outcomes and more easily prevent adverse effects.

The present study indicated that escalated irinotecan dose can help achieve the optimal values for cost-effective incremental costs of care in Taiwan. Few studies have analyzed treatment with different irinotecan doses combined with bevacizumab. Obradovic et al. divided patients with mCRC into those with or without specific *UGT1A1* genotypes (26); however, they did not compare patients who did not undergo genotyping (administered the fixed-dose regimen) and patients who underwent *UGT1A1* genotyping (administered escalated-dose regimen). If patients with UGT1A1*1/*28 and UGT1A1*1/*1 were administered an escalated dose of irinotecan and those with UGT1A1*28/*28 were given a reduced irinotecan dose, the overall total medical direct costs may be reduced. Butzke et al. performed a sensitivity analysis and discovered that a *UGT1A1* test before irinotecan chemotherapy and medical expenses were critical factors determining costs (27). Gold et al. conducted a sensitivity analysis and revealed that only when irinotecan dose adjustment achieved 98.4% of the effectiveness of the full dose, the DNA test can be continued (28). When ICUR was US$ 100,073 per QALY and WTP was US$ 100,000, *UGT1A1* genotyping test remained the most optimal choice before administering irinotecan. Therefore, treatment in the study group resulted in fewer disease recurrences, and thus, an ICUR decline when additional costs after disease recurrences increased. Given this, the escalated dose of irinotecan will be cost-effective compared with the recommended dose of irinotecan when combined with other costlier treatment strategies in mCRC patients. To enable decision making for tailored therapy, before administering irinotecan, different doses should be determined according to the *UGT1A1* genotype, thereby achieving more favorable cost-utility.

The following limitations of this study must be acknowledged. First, this was not a randomized study. At baseline, the study group (escalated dose group) had higher mean scores for FACT-C social/family wellbeing and BAI than the control group (recommended dose group). Therefore, the magnitude of QOL improvements obtained by different doses of irinotecan plus bevacizumab may have been underestimated. Nonetheless, the potential bias would not have changed our result that the study group seemed to achieve more cost-effectiveness than the control dose group. Second, we did not include costs involved in the nonhealthcare sector or from the societal perspective, including productivity loss and out-of-pocket expenses. Third, repeated measures of QOL outcomes were limited to 2 years. Basic symptoms of pain, neuropathic pain, and chemicals were not assessed, which is a noted limitation of the quality of life assessment in this study. Further randomized clinical trials are needed to corroborate the benefit on clinical outcomes, additional costs other than healthcare costs including survival, and overall societal impact.

The improved effectiveness in the study group was corroborated by evidence of significant improvements in QOL outcomes from posttherapeutic 6th month to posttherapeutic 2nd year. Nevertheless, we suggest that before the introduction of irinotecan treatment, patients with mCRC undergo *UGT1A1* genotyping to help determine the appropriate irinotecan dose, thereby achieving higher cost-utility and better medical resource allocation. Further scientific research is needed to determine what doses achieve the most desirable therapeutic effects in different UGT1A1 genotypes, which would translate into improved QOL after such a burdensome treatment of the underlying disease. Our results may serve as potential references for health departments, academic units, and medical supply units to treat mCRC.

## Data Availability Statement

The raw data supporting the conclusions of this article will be made available by the authors, without undue reservation.

## Ethics Statement

The studies involving human participants were reviewed and approved by the institutional ethics committee of our hospital (KMUHIRB-(EI)-20150147). The patients/participants provided their written informed consent to participate in this study.

## Author Contributions

H-YS, H-LT, and J-YW had full access to all the data in the study take responsibility for the integrity of the data and the accuracy of the data analysis. Concept and design: H-YS, H-LT, and J-YW. Acquisition, analysis, or interpretation of data: All authors. Drafting of the manuscript: H-YS, H-LT, and J-YW. Critical revision of the manuscript for important intellectual content: All authors. Statistical analysis: H-YS. Obtained funding: JYW. Supervision: H-YS, H-LT, and J-YW. All authors contributed to the article and approved the submitted version.

## Funding

This work was supported by grants through funding from the Ministry of Science and Technology (MOST 109-2314-B-037-035, MOST 109-2314-B-037-040, MOST 109-2314-B-037-046-MY3, MOST110-2314-B-037-097) and the Ministry of Health and Welfare (MOHW109-TDU-B-212-134026, MOHW109-TDU-B-212-114006, MOHW110-TDU-B-212-1140026) and funded by the health and welfare surcharge of on tobacco products, and the Kaohsiung Medical University Hospital (KMUH110-0R37, KMUH110-0R38, KMUH110-0M34, KMUH110-0M35, KMUH110-0M36, KMUHSA11013, KMUH-DK(C)110010, KMUH-DK(B)110004-3) and KMU Center for Cancer Research (KMU-TC109A04-1) and KMU Center for Liquid Biopsy and Cohort Research Center Grant (KMU-TC109B05) and KMU Office for Industry-Academic Collaboration (S109036), Kaohsiung Medical University. In addition, this study was supported by the Grant of Taiwan Precision Medicine Initiative, Academia Sinica, Taiwan, R.O.C.

## Conflict of Interest

The authors declare that the research was conducted in the absence of any commercial or financial relationships that could be construed as a potential conflict of interest.

## Publisher’s Note

All claims expressed in this article are solely those of the authors and do not necessarily represent those of their affiliated organizations, or those of the publisher, the editors and the reviewers. Any product that may be evaluated in this article, or claim that may be made by its manufacturer, is not guaranteed or endorsed by the publisher.
